# Delivering relational continuity of care in light of recent GP contract changes

**DOI:** 10.3399/BJGP.2025.0199

**Published:** 2025-07-22

**Authors:** Molly Dineen, Serge Engamba, Nada F Khan, Kate Sidaway-Lee, Polly Duncan, Jessica Watson, Philip Evans, Denis Pereira Gray

**Affiliations:** 1 Centre for Academic Primary Care, University of Bristol, Bristol, UK; 2 Exeter Collaboration for Academic Primary Care (APEx), University of Exeter, Exeter, UK; 3 Exeter Collaboration for Academic Primary Care (APEx), University of Exeter, Exeter, UK; and BJGP Associate Editor; 4 St Leonard's Practice, Exeter, UK; 5 Centre for Academic Primary Care, University of Bristol, Bristol, UK; and Chair of the Primary care Academic CollaboraTive (PACT); 6 Exeter Collaboration for Academic Primary Care (APEx), University of Exeter, Exeter, UK; and Research Consultant, St Leonard's Practice, Exeter, UK; 7 St Leonard's Practice, Exeter, UK; and Emeritus Professor, University of Exeter, Exeter, UK

Relational continuity of care, described as the ongoing interpersonal and therapeutic relationship built up between a patient and a specific GP over time, benefits patients, GPs, and the healthcare system. Its advantages are well-evidenced and wide reaching but pertinently, continuity of care is associated with reduced patient mortality, increased GP efficiency, and reduced healthcare system costs.^1^

Other forms of continuity include informational continuity (readily available access to patient information throughout the healthcare system) and managerial continuity (handovers of care between professionals or teams).^2^ However, neither can replace the increased trust, empathy, and understanding that comes with seeing a regular GP.^3^

“*Measuring continuity means that practices can then focus on improving it, whether that is for all patients, or for a particular patient group. There are a range of strategies that can be implemented by practices looking to improve relational continuity of care.*”

Relational continuity of care benefits the majority of patients, but there are groups that are considered to need it the most, including: older adults, frequent attenders, those with multiple long-term conditions, mental health conditions, and patients from vulnerable populations (for example, those socially or economically disadvantaged).^4^ Although the benefits of continuity have been demonstrated in these groups, there is limited evidence to verify that they specifically benefit more than others. What is evident is that relational continuity is lower among underserved populations, including ethnic minority groups^5^ and those in the most deprived areas,^2^ thus creating an ‘inverse continuity law’ where those that may need it the most, are least likely to receive it.

Continuity is key to delivering efficient and effective primary health care worldwide and, with increasing demands on UK general practice, its importance has rightly been recognised by policymakers in the latest iterations of NHS GP contracts in both England and Wales.^6,7^ In England, this is with a particular focus on identifying those patients who are likely to benefit the most.

## What do the new GP contracts say (and not say) about continuity of care?

Announced on 28 February 2025, NHS England’s GP contract for 2025–2026 includes changes that will encourage practices to consider the continuity of care that they deliver^6^. This is a welcome change in direction from previous contracts which have prioritised immediate access to appointments, often at the expense of continuity and to the detriment of at-risk populations^8^.

There is now an increasing shift towards community-based, GP-led care, with additional funding to support the employment of GPs and investment into the Advice and Guidance Service (a digital communication channel which enables GPs to access specialist advice while managing patients in the community). There is also the opportunity for practices to declare their organisational values with a patient charter, although how these standards could be used to promote continuity is not made explicit. What is stated explicitly is that Primary Care Networks will be incentivised to risk stratify their patients and identify those that would benefit the most from continuity of care. A key missing detail is whether this specifically relates to relational continuity, rather than managerial or informational continuity, which the contract also aims to improve with shared patient records.

What the contract does not yet offer is any guidance or incentive for practices to deliver relational continuity of care to the patients that have been identified, nor is there any requirement to measure or evaluate the continuity that is already being delivered. There is also a danger that this directive is creating work to identify patients in need of continuity, rather than acknowledging that the majority of patients benefit from continuity of care and focusing on how this can be achieved. Concerningly, the contract also contains several changes which might adversely impact relational continuity of care, including the increasing shift towards online access. Although working in this way might create opportunities to book patients in with their regular GP, patients most in need of continuity are likely to comprise the group that have the greatest difficulties with online access.^9^ Going forward, we need more evidence on the associations between different access models and continuity of care to inform these decisions.

The Welsh GP contract offers a more convincing step in the right direction. Having been the source of lengthy negotiation and debate, the 2024–2025 Welsh General Medical Services Contract^7^ has also creditably demonstrated a commitment to providing patients with high quality, personalised care. Most significantly, there is a proposal to incentivise practices to complete a quality improvement project on continuity of care, as part of the Quality Improvement Framework. This decision recognises the importance of measuring continuity of care at a practice level and once finalised, will provide practices with the support, resource, and funding to do so. This is an essential first step to delivering and improving the continuity that is delivered.

## So, how *do* we deliver and improve relational continuity of care?

We are firm that ‘if you don’t measure it, you can’t manage it’,^10^ and that the first step to improving relational continuity is to incentivise practices to measure it. The Welsh Government has recognised that an important drawback is that most practices in the UK do not currently measure their continuity, despite the existence of several validated measures (for example, the Usual Provider of Care Index [UPC] or the St Leonard’s Index of Continuity of Care [SLICC]). A few tools have been developed to capture these measures in practice (for example, One Care’s Continuity of Care Toolkit, St Leonard’s SLICC template or the University of Bristol’s Continuity of Care Calculator), however they are all limited to certain clinical settings or systems and require some technical expertise. There is therefore a strong need for more broadly applicable, integrated, and user-friendly options. We hope that clinical system suppliers will consider integrating these evidence-based continuity measures into their software to enable quick and easy practice-based measurement, and that policy makers will make this a priority.

Measuring continuity means that practices can then focus on improving it, whether that is for all patients, or for a particular patient group. There are a range of strategies that can be implemented by practices looking to improve relational continuity of care. In fact, a recent review of interventions piloted in UK general practice identified several methods, which are summarised in Box 1 .^11^

**Box 1.** A summary of the clinical interventions piloted in UK general practices to improve relational continuity of care

Clinical interventions
Assigning patients to named GPs, including the use of personal lists
Changing booking processes to prioritise continuity of care
Offering patients with multimorbidity a comprehensive 6-monthly review with the same clinician
Patient profiling to identify those that will benefit from continuity of care the most
Introduction of digital technology to enhance continuity of care (for example, online consultations and digital prompts/reminders)
Facilitating follow-up appointments with the same GP (for example, after acute illness or with test results)
Increasing appointment capacity (for example, by extending hours or using acute hubs to free up GPs to deliver continuity)

There is more work to be done to understand the most effective strategies, including for those most in need, in future research.

## Where to begin?

In light of this discussion about the importance of measuring continuity of care, in early 2026, the Primary care Academic CollaboraTive (PACT)^12^ aims to launch their fourth national study. PACT is a collaborative research network of professionals working in primary care and aims to support members to collect meaningful data in their own general practice settings. In this future study, which has not yet been launched, PACT will provide resources to help participants measure the continuity of care in their practices and will use clinician surveys to explore the associations between relational continuity and practice systems/access models. Participants will receive benchmarked practice reports, enabling them to compare their continuity metrics to other practices across the UK, including those serving similar populations and those with similar and different models of care. This initiative not only aims to enhance our understanding of how to improve continuity of care across various practice settings but also aims to support practices with the first step towards delivering higher quality care to patients.

We hope that future iterations of the GP contracts will build upon this evidence and commit to supporting practices with the funding and resource required to evaluate and improve continuity of care for all patients, while maintaining access. Promoting the widespread adoption of relational continuity is essential to ensuring consistent and high-quality patient care nationwide.
